# Field-Effect Capacitors Decorated with Ligand-Stabilized Gold Nanoparticles: Modeling and Experiments

**DOI:** 10.3390/bios12050334

**Published:** 2022-05-13

**Authors:** Arshak Poghossian, Tobias Karschuck, Patrick Wagner, Michael J. Schöning

**Affiliations:** 1 MicroNanoBio, 40479 Düsseldorf, Germany; 2 Institute of Nano- and Biotechnologies, FH Aachen, 52428 Jülich, Germany; karschuck@fh-aachen.de; 3 Laboratory for Soft Matter and Biophysics, KU Leuven, 3000 Leuven, Belgium; patrickhermann.wagner@kuleuven.be; 4 Institute of Biological Information Processing (IBI-3), Forschungszentrum Jülich, 52425 Juelich, Germany

**Keywords:** electrolyte-insulator-semiconductor capacitors, field-effect sensor, gold nanoparticles, capacitive model, nanoparticle coverage, aminooctanethiol

## Abstract

Nanoparticles are recognized as highly attractive tunable materials for designing field-effect biosensors with enhanced performance. In this work, we present a theoretical model for electrolyte-insulator-semiconductor capacitors (EISCAP) decorated with ligand-stabilized charged gold nanoparticles. The charged AuNPs are taken into account as additional, nanometer-sized local gates. The capacitance-voltage (*C*–*V*) curves and constant-capacitance (ConCap) signals of the AuNP-decorated EISCAPs have been simulated. The impact of the AuNP coverage on the shift of the *C*–*V* curves and the ConCap signals was also studied experimentally on Al–p-Si–SiO_2_ EISCAPs decorated with positively charged aminooctanethiol-capped AuNPs. In addition, the surface of the EISCAPs, modified with AuNPs, was characterized by scanning electron microscopy for different immobilization times of the nanoparticles.

## 1. Introduction

Biosensing technologies using a field-effect device (FED) platform have received immense interest in recent years, motivated by the need for miniaturized, low-cost, and highly sensitive and selective point-of-care diagnostic devices capable of fast, label-free, real-time and multiplexed detection of analyte molecules. Typically, silicon-based biologically sensitive FEDs (BioFEDs) consist of an electrolyte-insulator-semiconductor (EIS) structure modified with receptors, which serves as basic transducer architecture for constructing a wide variety of chemical sensors and biosensors (see, e.g., recent reviews [1,2,3,4,5,6,7,8,9]). For instance, BioFEDs were developed for the analysis of metabolites [10,11,12,13], antibiotics [13,14], and for the label-free detection of charged molecules (nucleic acids [15,16,17,18], protein biomarkers [19,20,21,22,23], polyelectrolytes [24,25]) and charged nano-objects (e.g., ligand-stabilized gold nanoparticles (AuNP)) [26,27], nanoparticle/molecule hybrids [28], virus particles [5,29,30,31]). The concept also enabled us to design enzyme-based logic gates, which mimic the functioning of electronic logic gates [32,33]. Furthermore, a multifunctional sensor chip was developed for the detection of both biochemical and physical parameters in liquids (temperature, flow velocity and direction, diffusion coefficient and liquid level) by using the same FED principle [34].

Recent progress in nanomaterials and nanotechnology has opened new horizons in BioFED development. Decoration of the BioFED surface with chemically, electrically, or magnetically tunable nanomaterials, such as AuNPs, oxide nanoparticles, magnetic beads, carbon nanotubes and virus-like particles, is considered as a very promising strategy for designing biosensors with enhanced performance. The benefits of these nanomaterials include a high surface-to-volume ratio, easy surface modification with various shell molecules for coupling of bioreceptors, a high and reproducible density of these receptors with orientational freedom for receptor-target interactions, an enhanced transport of target molecules to the nanomaterial surface, and the facile integration with macroscopic transducers [35,36,37]. All these factors result in an improvement of the biosensor performance. This holds, for example, for EIS capacitors (EISCAP), ion-sensitive field-effect transistors (ISFET) and silicon nanowire transistors decorated with AuNPs to study DNA-hybridization and denaturation [16], and to detect protein kinase A [38], hemoglobin-A1c (HbA1c) [39] and prostate specific antigen [40] biomarkers. The approach was also used to study the streptavidin–biotin interaction [41], small proteins [28] and the electrostatic adsorption of polyelectrolytes [27,28]. It has been shown that the modification of a light-addressable potentiometric sensor with a carbon nanotube/enzyme layer significantly improves the sensitivity of the biosensor [42]. Furthermore, an EISCAP covered with magnetic nanoparticles terminated with carboxylic acid was used to develop a biosensor for ochratoxin A antigens [43]. Finally, a highly sensitive penicillin biosensor with a long shelf-life was engineered by modifying the EISCAP surface with nanotubular *tobacco mosaic virus* particles acting as enzyme nanocarriers [44].

To our knowledge, detailed theoretical models for nanoparticle-decorated FEDs are missing so far. A simplified model for the interfacial potential changes, induced by the electrostatic adsorption of charged molecules onto AuNP-modified EISCAPs, was proposed in [28]. A refined, theoretical description and simulation of BioFEDs decorated with various nanoparticles will allow to understand the signal generation at a more fundamental level. In turn, this will lead to optimized devices and will enable to predict their technological potential. In this work, we establish a capacitive model for the AuNP-decorated EISCAP. The capacitance-voltage (*C*–*V*) curves of the EISCAPs are simulated as a function of the AuNP coverage. In addition, EISCAPs decorated with positively charged aminooctanethiol-capped AuNPs (AOT-AuNP) were prepared and characterized electrochemically in the *C*–*V*- and constant-capacitance (ConCap) modes. The surface of these EISCAPs was physically characterized by scanning electron microscopy (SEM) for different immobilization times of the nanoparticles to correlate the AuNP coverage with the shift of the *C*–*V* curves and ConCap data.

## 2. Modeling and Simulation of AuNP-Decorated EISCAPs

**Figure 1:**
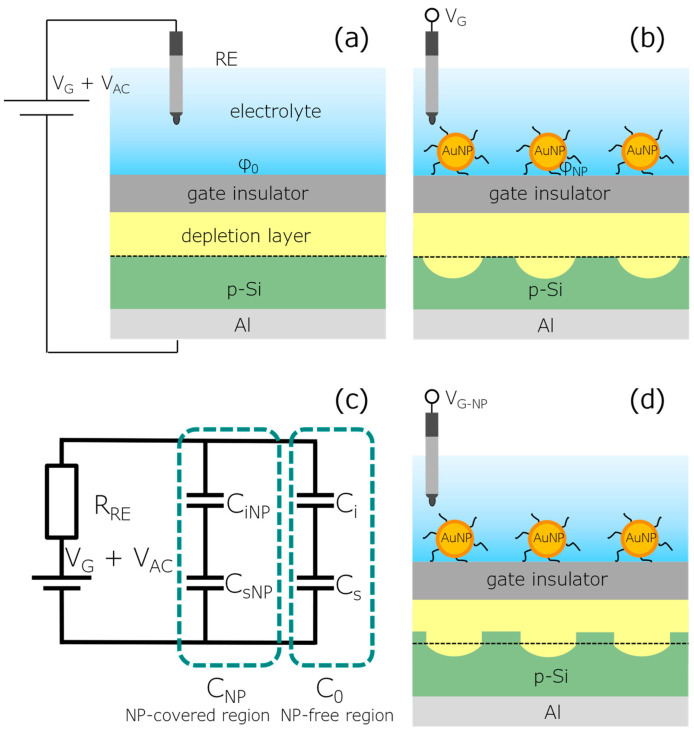
Schematic structure and shape of the depletion layer in p-Si for a bare EISCAP (**a**) and an EISCAP decorated with ligand-stabilized positively charged AuNPs (**b**); (**c**) electrical equivalent circuit of an AuNP-decorated EISCAP; and (**d**) local changes in the width of the depletion layer at the gate voltage (*V*_G-NP_), which is applied to the AuNP-decorated EISCAP in the ConCap mode to keep the working capacitance constant. *R*_RE_: resistance of the reference electrode; *V*_G_: gate voltage; *V*_AC_: alternating current voltage; *C*_iNP_, *C*_sNP_ and *C*_NP_: gate-insulator, space-charge and total capacitances in an AuNP-covered region, respectively; *C*_i_, *C*_s_ and *C*_0_ are the corresponding parameters for AuNP-free regions; *φ*_0_ and *φ*_NP_: gate insulator-electrolyte interfacial potential in the AuNP-free and AuNP-covered regions.

Figure 1 shows the structure and shape of the depletion layer in p-Si for a bare EISCAP (a) and an EISCAP decorated with ligand-stabilized positively charged AuNPs (b). Due to electrostatic and other interactions between the AuNPs and the EISCAP surface, as well as inter-particle repulsion, the immobilized AuNPs usually do not form densely packed layers (i.e., the EISCAP surface is partially covered with NPs). Hence, AuNPs can be considered as additional, nanometer-sized local gates, whose potential can be tuned by the intrinsic charge of attached target biomolecules. Figure 1b illustrates schematically how the depletion layer in the p-Si is modulated locally by the presence of the charged NPs. The effect of immobilizing AuNPs and of coupling charged molecules to the AuNPs surface is similar to applying an additional voltage to the local gates. In a first approach, we assume that the distance between the AuNPs is sufficiently large to prevent possible overlapping of the local depletion regions in the Si due to the fringing effect [45]. Hence, the AuNP-decorated EISCAP can be considered as consisting of two regions: (i) an AuNP-covered region with a surface coverage of *n* = *A*_NP_/*A* and an interfacial potential of *φ*_NP_, and (ii) an AuNP-free region with a surface area of *A*_0_ = (1 − *n*)*A* and an interfacial potential of *φ*_0_. Here, *A*_NP_ is the surface area covered with AuNPs and *A* is the whole surface area of the EISCAP in contact with the electrolyte.

The simplified electrical equivalent circuit of the AuNP-decorated EISCAP is depicted in Figure 1c. The resistances of the electrolyte, bulk Si and Al-Si rear-side contact are typically much smaller than the resistance of the reference electrode (*R*_RE_) and are therefore not included in the equivalent circuit. In addition, for typical thicknesses of the gate insulator materials (10–100 nm), measuring frequencies of below 1 kHz and an ionic strength of the electrolyte of >0.1 mM, the double-layer capacitance can also be neglected, see refs. [46,47]. Hence, the equivalent capacitance (*C*_eq_) of the AuNP-modified EISCAP is given by:

(1) $$C_{\mathrm{eq}}\;{=\;C}_{\mathrm{NP}}\;{+\;C}_{0}\;{=\;C}_{\mathrm{iNP}}C_{\mathrm{sNP}}/\left(C_{\mathrm{iNP}}\;+\;C_{\mathrm{sNP}}\right){\;+\;C}_{i}C_{s}/{(C}_{i}{\;+C}_{s})$$

where *C*_NP_ is the total capacitance of the AuNP-covered region consisting of the gate-insulator (*C*_iNP_ = *ε*_i_*An*/*d*_i_) and space-charge (*C*_sNP_ = *ε*_s_*An*/*w*_NP_) capacitances in series. *C*_0_ is the total capacitance of the AuNP-free region and combines the gate-insulator (*C*_i_ = *ε*_i_*A*(1 − *n*)/*d*_i_) and space-charge (*C*_s_ = *ε*_s_*A*(1 − *n*)/*w*_s_) capacitances in series. The symbols *ε*_i_ and *ε*_s_ denote the permittivity of the gate insulator and semiconductor, *w*_NP_ and *w*_s_ are the widths of the space-charge regions in the semiconductor with and, respectively, without, AuNP coverage.

The EISCAPs are conveniently characterized by using the *C*–*V* and/or ConCap measurement mode. To determine the impact of the AuNP coverage on the *C*–*V* curve of the AuNP-decorated EISCAPs, we define the equivalent capacitance in the accumulation, depletion and inversion regions. For the p-type Si, in the accumulation region (at gate voltages of *V*_G_ < 0), *C*_iNP_ << *C*_sNP_ and *C*_i_ << *C*_s_. Hence, the equivalent capacitance of the AuNP-decorated EISCAP in the accumulation region (*C*_eq-acc_) can be obtained from Equation (1) as:

(2) $$C_{\mathrm{eq}\text{-}\mathrm{acc}}\;=\;C_{\mathrm{iNP}}\;+\;C_{i}\;=\;\varepsilon_{i}A/d_{i}$$

It is well known, that the space-charge capacitance in the Si, and consequently the overall capacitance of the EISCAP sensor, is affected by the potential changes at the gate-insulator/electrolyte interface due to adsorption or binding of charged molecules or particles. This results in a shift of the *C*–*V* curve along the voltage axis in the depletion region, see, e.g., ref. [6]. The width of the depletion layer in the Si underneath the AuNP-covered or AuNP-free regions can be derived from the expression for the space-charge width (*w*_MOS_) of a MOS (metal-oxide-semiconductor) capacitor [48]:

(3) $$w_{\mathrm{MOS}}\;=\;(\varepsilon_{s}^{2}/C_{i0}^{2}\;+\;{2\varepsilon }_{s}V_{G}/{qN}_{a}{)}^{1/2}-\varepsilon_{s}/C_{i0}$$

where *C*_i0_ = *ε*_i_/*d*_i_ is the insulator capacitance per unit area, *N*_a_ is the density of ionized acceptors and *q* is the elementary charge. To obtain *w*_NP_ or *w*_s_, *V*_G_ in Equation (3) should be replaced with (*V*_G_ − *V*_fbNP_) or (*V*_G_ − *V*_fb_), respectively, where *V*_fbNP_ and *V*_fb_ are the flat-band voltages of the EISCAP in the AuNP-covered and AuNP-free region. *V*_fbNP_ and *V*_fb_ can be expressed as [46]:

(4) $$V_{\mathrm{fb}\;}=\;V_{\mathrm{ip}}\;-\;\varphi_{0}$$

(5) $$V_{\mathrm{fbNP}}\;=\;V_{\mathrm{ip}}\;-\;\varphi_{\mathrm{NP}}\;=\;V_{\mathrm{ip}}\;-\;(\varphi_{0}\;\pm \;\Delta \varphi )$$

with

(6) $$V_{\mathrm{ip}}\;=\;E_{\mathrm{ref}}\;+\;\chi_{\mathrm{sol}}\;-\;\phi_{s}/q\;+\;(Q_{i}\;+Q_{\mathrm{ss}}{)/C}_{i}$$

where Δ*φ* is the interfacial potential change induced by the coupled ligand-stabilized charged AuNPs, *V*_ip_ represents a group of AuNP-independent potentials, *E*_ref_ is the potential of the reference electrode relative to vacuum, *χ*_sol_ is the surface-dipole potential of the solvent, *ϕ*_s_ is the silicon electron work function, and *Q*_i_ and *Q*_ss_ are the charges located in the oxide and the surface and interface states, respectively. Equations (3)–(6) allow us to derive the following expressions for *C*_sNP_ and *C*_s_:

(7) $$C_{\mathrm{sNP}}=\frac{\varepsilon_{s}An}{w_{\mathrm{NP}}}=\frac{An}{{[1/C_{i0}^{2}+2\left(V_{G}-{\;V}_{\mathrm{ip}}\;+\;\varphi_{0}\;\pm \;\Delta \varphi \right)/{q\varepsilon }_{s}N_{a}]}^{1/2}-1/C_{i0}}$$

(8) $$C_{s}=\frac{\varepsilon_{s}\;A(1-n)}{w_{s}}=\frac{A(1-n)}{{[1/C_{i0}^{2}+2\left(V_{G\;}-{\;V}_{\mathrm{ip}}+\varphi_{0}\right)/{q\varepsilon }_{s}N_{a}]}^{1/2}-1/C_{i0}}$$

The equivalent capacitance of the AuNP-modified EISCAP in the depletion region (*C*_eq-dep_) is obtained by substituting Expressions (7) and (8), *C*_iNP_ = *ε*_i_*An*/*d*_i_ = *AnC*_i0_ and *C*_i_ = *ε*_i_*A*(1 − *n*)/*d*_i_ = *A*(1 − *n*)*C*_i0_ into Equation (1):

(9) $$C_{\mathrm{eq}\text{-}\mathrm{dep}}=\frac{An}{{[1/C_{i0}^{2}\;+2\left(V_{G}-V_{\mathrm{ip}}+\varphi_{0}\;\pm \;\Delta \varphi \right)/{q\varepsilon }_{s}N_{a}]}^{1/2}}+\frac{A(1-n)}{{[1/C_{i0}^{2}+2\left(V_{G\;}-{\;V}_{\mathrm{ip}}+\varphi_{0}\right)/{q\varepsilon }_{s}N_{a}]}^{1/2}}$$

The analysis of Equation (9) reveals that, at a constant gate voltage *V*_G_, the equivalent capacitance in the depletion region (*C*_eq-dep_) is, among others, a function of the AuNP coverage (*n*) and an interfacial potential change (Δ*φ*) caused by the coupling of charged AuNPs on the EISCAP surface. The direction of this potential change depends on the charge sign of ligand-stabilized AuNPs. A simplified relation between Δ*φ* and the effective AuNP charge (*Q*_NP_) can be expressed as [28]:

(10) $${\Delta \varphi =N}_{\mathrm{NP}}Q_{\mathrm{NP}}/C_{d}\;$$

where *N*_NP_ is the density of AuNPs and *C*_d_ is the double-layer capacitance per surface area in the AuNP-covered region. Note that due to the counterion-screening effect, the effective charge of AuNPs and, therefore, the interfacial potential changes will also depend on the ionic strength of the measurement solution. While Equation (10) describes a simplified approximation, it indicates nonetheless that *C*_eq-dep_, and therefore the *C*–*V* curves of the EISCAP, are gated by the effective charge of the decorated AuNPs.

To obtain the expression for the equivalent capacitance of the AuNP-decorated EISCAP in the inversion range, we assume that by strong inversion, the depletion layer in both the NP-covered and NP-free regions will reach its maximum width, *w*_m_ [48]:

(11) $$w_{m}=[\frac{{4\varepsilon }_{s}kT\;\ln\left(N_{a}/n_{i}\right)}{q^{2}N_{a}}{]}^{1/2}$$

where *k* is the Boltzmann constant, *T* is the absolute temperature, and *n*_i_ is the electron density in the intrinsic semiconductor. Correspondingly, the high-frequency inversion capacitances of the NP-covered (*C*_invNP_) and NP-free (*C*_inv_) regions will reach their minimum:

(12) $$C_{\mathrm{sNP}}{=C}_{\mathrm{invNP}}{=\varepsilon }_{s}An/w_{m}\;\mathrm{and}\;C_{s}{=C}_{\mathrm{inv}}{=\varepsilon }_{s}A(1-n)/w_{m}$$

The equivalent capacitance of the NP-decorated EISCAP in the inversion range (*C*_eq-inv_) can be obtained by substituting expressions (12) into Equation (1):

(13) $$C_{\mathrm{eq}\text{-}\mathrm{inv}}=C_{\mathrm{iNP}}C_{\mathrm{invNP}}/\left(C_{\mathrm{iNP}}+C_{\mathrm{invNP}}\right)+C_{i}C_{\mathrm{inv}}/{(C}_{i}+C_{\mathrm{inv}})$$

Considering that usually *C*_iNP_ >> *C*_invNP_ and *C*_i_ >> *C*_inv_, Equation (13) can be simplified as:

(14) $$C_{\mathrm{eq}\text{-}\mathrm{inv}}=\frac{\varepsilon_{s}A}{w_{m}}{=\varepsilon }_{s}qA[\frac{N_{a}}{{4\varepsilon }_{s}kT\;\ln\left(N_{a}/n_{i}\right)}{]}^{1/2}$$

The combination of Equations (2), (9) and (13) gives the complete course of the *C–V* curve of the AuNP-decorated EISCAP. We simulated *C*–*V* curves of the AuNP-decorated EISCAP with different AuNP coverages using Equations (2), (9) and (13) in Python 3.9 (Python Software Foundation). The simulation parameters were: *ε*_i_ = *ε*_0_*ε*_ir_, *ε*_0_ = 8.854 × 10^−12^ F/m, *ε*_ir_ = 3.9 (SiO_2_), *d*_i_ = 35 nm, *ε*_s_ = *ε*_0_*ε*_sr_, *ε*_sr_ = 11.7 (Si), *n*_i_ = 1.5 × 10^10^ cm^−3^, *N*_a_ = 2.76 × 10^15^ cm^−3^ (that corresponds to the resistivity of a Si wafer of 5 Ω∙cm), *q* = 1.6 × 10^−19^ C, *k* = 1.38 × 10^−23^ J/K, *T* = 300 K, *A* = 0.5 cm^2^, *V*_ip_ = 0, *φ*_0_ = −10 mV, *φ*_NP_ = 30 mV, *n* = 0, 0.25, 0.5, 0.75, and 0.9.

**Figure 2:**
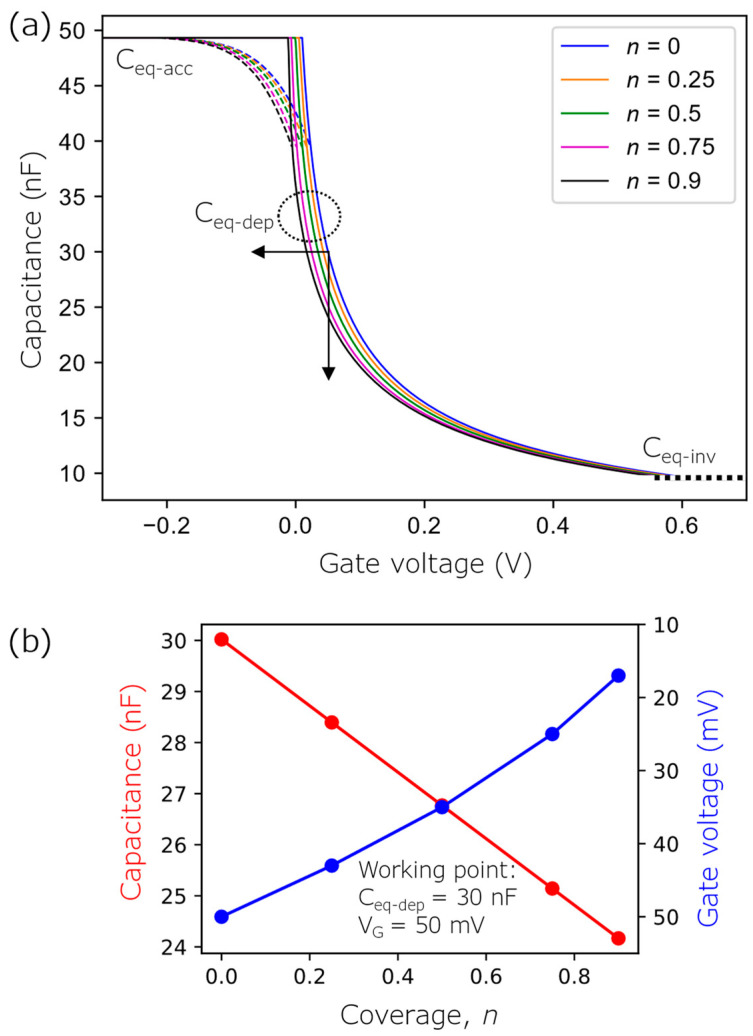
(**a**) Simulated *C*–*V* curves of a bare, p-type EISCAP and an EISCAP decorated with positively charged AuNPs with different coverages *n* (from 0.25 to 0.9). The dashed curves illustrate the expected course of the overall equivalent capacitance of the EISCAP in the transition region from depletion to accumulation. (**b**) Capacitance changes (at a constant gate voltage of *V*_G_ = 50 mV) and voltage shifts (at a constant capacitance of *C*_eq-dep_ = 30 nF) as a function of the AuNP coverage, evaluated from the *C*–*V* curves.

Figure 2a illustrates the simulated *C*–*V* curves of a p-type bare EISCAP and an EISCAP decorated with positively charged AuNPs with different coverages *n*. At a constant gate voltage, the existence of positively charged AuNPs on the EISCAP surface leads to an increasing width of the depletion layer in the Si underneath AuNP-covered regions; the corresponding space-charge capacitance in the Si will decrease. As a result, the overall capacitance of the AuNP-decorated EISCAP will decrease as well, resulting in a shift of the original *C*–*V* curve (*n* = 0) towards less positive gate voltages (or more negative voltages). By increasing the coverage of positively charged AuNPs, the magnitude of these voltage shifts is increasing. The capacitance changes (at a constant gate voltage of *V*_G_ = 50 mV) and voltage shifts (at a constant capacitance of *C*_eq-dep_ = 30 nF) as a function of the AuNP coverage, evaluated from the *C*–*V* curves, are shown in Figure 2b.

In addition to the dependency of the *C*–*V* curve on the AuNP coverage and interfacial potential changes, it is very interesting to determine the ConCap signal changes generated by the coupling of charged AuNPs. In contrast to the *C*–*V* method, the ConCap measuring mode allows us to study the dynamic behavior of the EISCAP sensor signal. It is widely accepted that in the ConCap mode, by setting the working capacitance at a fixed value using a feedback control circuit, the interfacial potential changes due to the adsorption/binding of charged molecules or nano-objects on the sensor surface can be directly recorded.

Hence, it is suggested that the recorded change in the ConCap signal is equal to the interfacial potential change. This statement is correct if the entire sensor surface is covered with charged molecules or nano-objects, i.e., when the coverage is *n* = 1. However, this is often not the case for NP-decorated BioFEDs: usually, due to the inter-particle repulsion and/or immobilization conditions, the sensor surface is only partially covered with charged NPs (i.e., *n* < 1). As a result, the recorded ConCap signal change will not be equal to the interfacial potential change, which occurs in the NP-covered region. Moreover, it will depend, among others, on the AuNP coverage. The gate voltage (*V*_G-NP_) in the ConCap mode, which should be applied to the AuNP-decorated EISCAP to keep the working capacitance (chosen from the depletion region of the *C*–*V* curve of the bare EISCAP) constant, can be obtained by using Equation (9) and the condition *C*_eq-dep_ = *C*_eq-dep_(*n* = 0), where *C*_eq-dep_(*n* = 0) is the equivalent capacitance of the non-modified EISCAP in the depletion region:

(15) $$\frac{n}{\sqrt{\frac{1}{C_{i0}^{2}}+\frac{{2(V}_{G\text{-}\mathrm{NP}}-V_{\mathrm{ip}}+\varphi_{\mathrm{NP}})}{{q\varepsilon }_{s}N_{a}}}}+\frac{1-n}{\sqrt{\frac{1}{C_{i0}^{2}}+\frac{{2(V}_{G\text{-}\mathrm{NP}}-V_{\mathrm{ip}}+\varphi_{0})}{{q\varepsilon }_{s}N_{a}}}}=\frac{1}{\sqrt{\frac{1}{C_{i0}^{2}}+\frac{{2(V}_{G}-V_{\mathrm{ip}}+\varphi_{0})}{{q\varepsilon }_{s}N_{a}}}}$$

**Figure 3:**
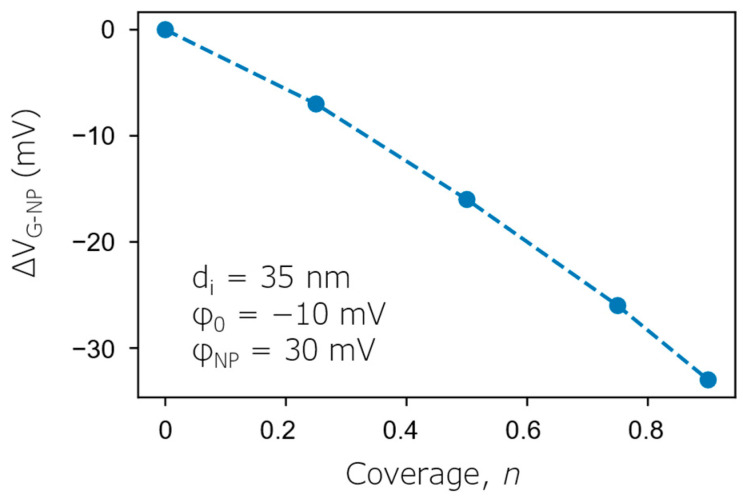
Calculated ConCap signal change (Δ*V*_G-NP_) of an AuNP-decorated EISCAP as a function of the AuNP coverage with *n* varying from 0.25 to 0.9.

Local changes in the width of the depletion layer in NP-covered and NP-free regions at the gate voltage (*V*_G-NP_) applied in the ConCap mode are sketched in Figure 1d. The additional gate voltage (Δ*V*_G-NP_ = *V*_G-NP_ − *V*_G_) in the ConCap mode (i.e., the ConCap signal change), which is applied to the AuNP-decorated EISCAP to keep the working capacitance constant, was calculated as a function of the AuNP coverage using Equation (15) and is shown in Figure 3. As can be seen, with increasing the AuNP coverage from *n* = 0.25 to *n* = 0.9, Δ*V*_G-NP_ increases from −7 mV to −33 mV. However, the Δ*V*_G-NP_ values are always smaller than the interfacial potential changes in the AuNP-covered region (Δ*φ* = *φ*_NP_ − *φ*_0_ = 40 mV), which is supported by the discussion above.

## 3. Experimental

### 3.1. Preparation of AuNPs

Gold nanoparticles covered with aminooctanethiol (AOT) were prepared from citrate-capped AuNP (Ct-AuNP) using the ligand-exchange reaction from citrate to aminooctanethiol. The negatively charged Ct-AuNPs were synthesized with the Turkevich method using the tetrachloroauric acid (HAuCl_4_) reduction reaction [49,50]. Citrate acts as the reducing agent and as stabilizing ligand to prevent AuNPs from aggregating. Briefly, 100 mL of a 0.25 mM HAuCl_4_·3H_2_O (hydrogen tetrachloraurate(III) trihydrate, Thermo Fisher, Bremen, Germany) solution was brought to boiling under continuous stirring. Then, 1 mL of a 500 mM Na_3_Ct (trisodium citrate dihydrate, Merck, Darmstadt, Germany) solution was added and boiling continued until the color changed from yellow to burgundy, indicating the formation of nanoparticles. The solution was kept boiling for an additional 5–10 min, then left to cool down to room temperature and stored at 4 °C.

The exchange of capping ligands on AuNPs from citrate to AOT was achieved by mixing 10 mL Ct-AuNP solution with 4 mL of 100 mM HCl and 400 μL of 2 mM AOT (8-amino-1-octanethiol hydrochloride in ethanol, Merck, Germany). Due to the higher binding affinity of the thiol groups to gold, the citrate molecules are completely replaced by AOT ligands [51]. After removing unbound AOT residues in the solution by centrifugation, the AOT-AuNPs were stored at 4 °C. For the AuNP loading experiments, the AOT-capped pellets were redispersed in 250 μL of 20 mM HEPES buffer (4-(2-hydroxyethyl)-1-piperazineethanesulfonic acid, Carl Roth, Karlsruhe, Germany) containing 20 mM NaCl, pH 3 (further referred to as AuNP solution), resulting in an AuNP concentration of 0.8 nM. For more details on the preparation of AOT-stabilized AuNPs, see refs. [27,51].

In order to verify the ligand exchange from negatively charged citrate to positively charged AOT, the zeta potential of the AuNPs was measured by electrophoretic light scattering using the Litesizer 500 (Anton Paar, Ostfildern, Germany). Measurements were conducted in citrate buffer of pH 6.5 (ionic strength: 4 mM) and HEPES buffer of pH 3 (ionic strength: 30 mM) for citrate- and AOT-capped AuNPs, respectively. As expected, the synthesized Ct-AuNPs were negatively charged (due to the carboxylic acid groups of the citrate molecules) with a zeta potential of −59 mV, while AOT-AuNPs were positively charged (due to the ammonium groups) with a zeta potential of ca. +35 mV. These results confirm a successful ligand-exchange reaction.

### 3.2. Fabrication of EISCAPs

The field-effect EISCAPs were fabricated from commercially available p-Si-SiO_2_ wafers (Siegert Wafer, Aachen, Germany) with a 30 nm SiO_2_ layer prepared by dry thermal oxidation of Si. After etching the rear-side SiO_2_ layer with hydrofluoric acid (HF, 5%), a 300 nm thick Al layer was deposited on the Si substrate by electron-beam evaporation as back-side contact layer and annealed under nitrogen atmosphere at 400 °C for 10 min. Then, the Al-p-Si-SiO_2_ wafer was cut into single EISCAP chips with a size of 1 cm × 1 cm, followed by cleaning the chips in an ultrasonic bath and drying with N_2_.

For the experiments, the EISCAP chip and the reference electrode (Ag/AgCl filled with 3 M KCl, Metrohm, Filderstadt, Germany) was mounted in a homemade measurement cell and connected to an impedance analyzer (Zahner Zennium, Zahner Elektrik, Kronach, Germany). The front side contact area of the EISCAP chip with the electrolyte solution was ca. 50 mm^2^.

### 3.3. Immobilization of AOT-Capped AuNPs onto the EISCAP Chip Surface

Before decorating the EISCAP surface with AOT-capped AuNPs, the EISCAP was mounted in the measurement cell and preconditioned in 0.33 mM PBS (phosphate buffered saline, pH 3) for at least 12 h. Thereafter, 40 µL of 0.8 nM AOT-capped AuNP solution was dropped on the chip surface and incubated for 0.5 h, 1 h and 2 h. After immobilization of AuNPs, the chips were rinsed three times with 1 mL of 0.33 mM PBS to remove unattached AuNPs, and dried in N_2_ gas. Two groups of AuNP-decorated EISCAP chips were prepared identically: three chips for the electrochemical characterization and three chips for SEM characterization. For SEM characterization, the AuNP-decorated EISCAP chips were removed from the measurement cell, rinsed with deionized water to remove salt residues and dried in a N_2_ stream.

## 4. Results and Discussions

### 4.1. Immobilization of AOT-Capped AuNPs onto the EISCAP Chip Surface

**Figure 4:**
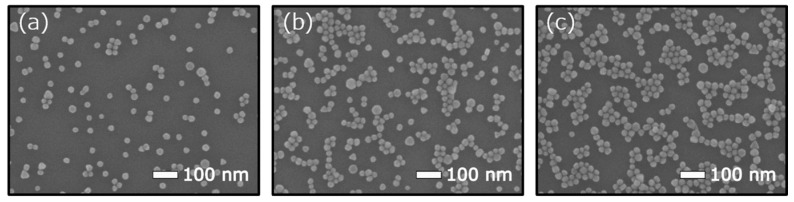
Representative SEM images of an EISCAP surface decorated with AOT-AuNPs with immobilization times of 0.5 h (**a**), 1 h (**b**) and 2 h (**c**).

SEM images of the AuNP-decorated EISCAP surface were taken with a high-resolution JSM-7800F Schottky field-emission scanning electron microscope (Jeol GmbH, Freising, Germany). An ImageJ-software macro [52] was utilized to determine the size of decorated AuNPs and to calculate their surface density and coverage from the SEM images (15 images were taken per EISCAP chip). Figure 4 shows representative SEM images of EISCAP surface decorated with AOT-AuNPs with immobilization times of 0.5 h, 1 h and 2 h (from left to right).

The core diameter of the AOT-AuNPs was found to be 28 ± 3 nm. In our previous work with the same preparation procedures, the number of AOTs per AuNP was estimated to be ≈8.8 × 10^3^ molecules [27]. As expected, the longer immobilization time significantly increases the particle density on the chips: We found (235 ± 6) × 10^8^ AOT-NPs per cm^2^ for 0.5 h of incubation which increases to (381 ± 15) × 10^8^ AOT/cm^2^ for 1 h and to (617 ± 19) × 10^8^ AOT/cm^2^ for 2 h. The maximum density of AOT-AuNPs achieved in this study is comparable to literature values for AuNP-decorated SiO_2_ surfaces [27,28,53,54]. The corresponding surface-coverage values were *n* = 0.12 (for 0.5 h incubation time, *n* = 0.22 (1 h) and *n* = 0.36 (2 h). Note, the adsorbed AuNPs on SiO_2_ surfaces withstood multiple uses and treatment steps. Even after multiple rinsing and drying procedures, we did not observe any significant decrease of the surface density of AuNPs evaluated from the SEM images.

### 4.2. Capacitance-Voltage Curves and Constant-Capacitance Signal of AuNP-Decorated EISCAPs

**Figure 5:**
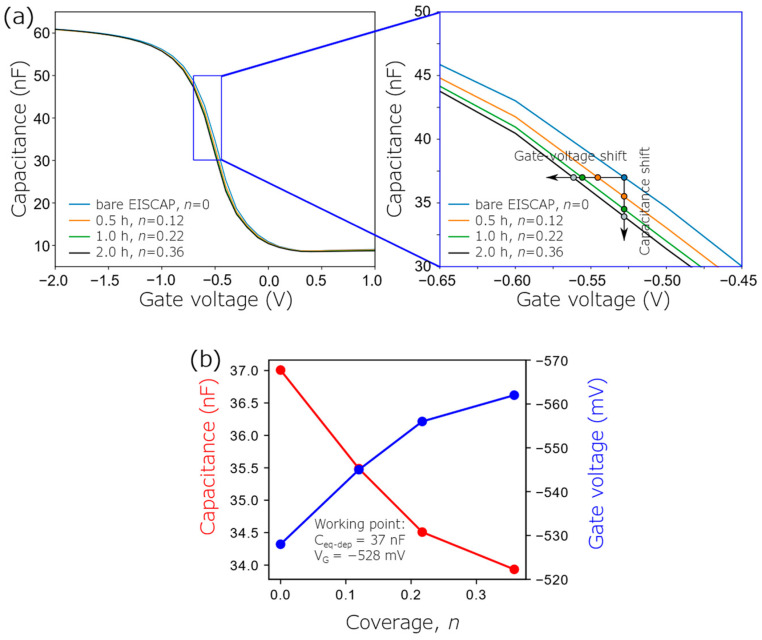
(**a**) Measured *C*–*V* curves of a bare EISCAP and an EISCAP decorated with positively charged AOT-AuNPs of different coverages *n* (for different times of immobilization between 0.5 and 2 h) with a zoomed graph of the depletion region. (**b**) Capacitance changes (at a constant gate voltage of −528 mV) and gate voltage shifts (at a constant capacitance of 37 nF) evaluated from the *C*–*V* curves as a function of the AuNP coverage.

**Figure 6:**
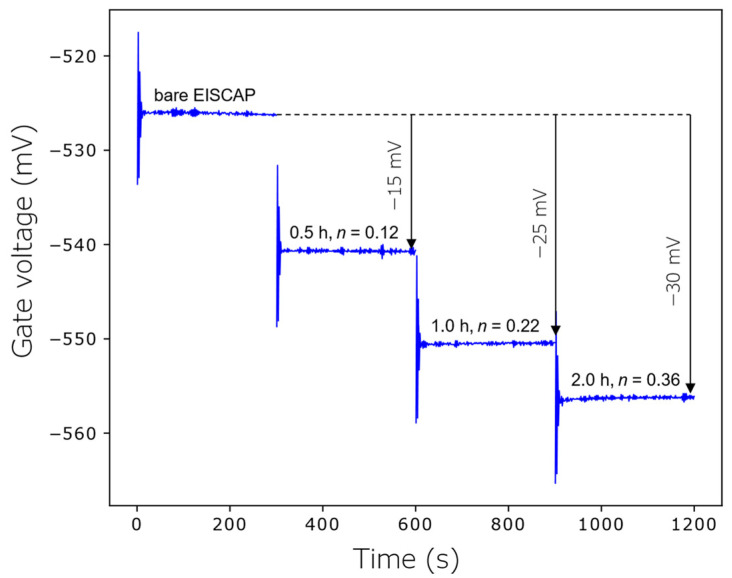
Dynamic ConCap signal change of the EISCAP decorated with positively charged AuNPs of different coverages *n*, corresponding to AuNP immobilization times of 0.5 h, 1 h and 2 h.

The EISCAPs were electrochemically characterized in the *C*–*V* and ConCap mode before and after immobilization of the AOTs for 0.5 h, 1 h and 2 h (see Figure 5 and Figure 6).

It is well known that in electrolyte solution, the charge associated with the biomolecules or NPs is screened or neutralized (in whole or part) by small counterions present in the solution. FEDs are able to detect charge/potential changes occurring directly at the gate surface or within the order of the Debye length from the surface, which is inversely proportional to the ionic strength of the solution. In order to reduce the impact of the Debye screening effect [55,56,57] and maximize the EISCAP signal amplitude, the measurements were conducted in a low ionic-strength (5 mM) solution (0.33 mM PBS, pH 3). In a measurement solution of pH 3, the terminal amino groups of the capped AOT molecules can be considered as fully protonated [51] (that may ensure highly charged AuNPs and therefore, large EISCAP signal), while the SiO_2_ surface is still slightly negatively charged (note that the pH_pzc_ (point of zero charge) of SiO_2_ is approximately 2.66–2.8 pH [58,59,60]).

The experiment was carried out in the following order: First, the *C*–*V* curves and the ConCap signal of the bare EISCAP were measured to define the baseline signal. Then, the EISCAP surface was decorated with AuNPs by immobilization of AOT-AuNPs for 0.5 h. After rinsing and drying, the AuNP-decorated EISCAP sensor was characterized again in the *C*–*V*- and ConCap modes. To assess the impact of the AuNP coverage on the EISCAP signal, these decoration- and measurement process steps were repeated for an additional 0.5 h (complete immobilization time: 1 h) and 1 h immobilization (complete immobilization time: 2 h).

Exemplary *C*–*V* curves of the bare and AuNP-decorated EISCAP with different AuNP-immobilization times and therefore, various AuNP coverages are presented in Figure 5. As expected, the decoration of the EISCAP surface with positively charged AOT-AuNPs shifts the original *C*–*V* curve of a non-modified EISCAP towards more negative voltages. The reason for this behavior of the *C*–*V* curves in case of a p-type EISCAP covered with positively charged AuNPs has been discussed in Section 2. The changes in the overall capacitance of the AuNP-decorated EISCAP (at a constant gate voltage *V*_G_ = −528 mV) and the amplitude of the voltage shifts (at a constant capacitance of 37 nF) evaluated from the *C*–*V* curves are depicted in Figure 5b. As predicted by the capacitive model described in Section 2, the overall capacitance of the EISCAP decreases with increasing AuNP coverage, while the amplitude of the gate-voltage shifts increases. A gate-voltage shift of −34 mV was recorded for the maximum AuNP coverage of *n* = 0.36, which is achieved in this study.

The results of ConCap-mode measurements are presented in Figure 6. Similar to the *C*–*V* curves, with increasing AuNP coverage, the ConCap signal shifts to more negative voltages; at the maximum AuNP coverage of *n* = 0.36, the signal shift of about −30 mV was recorded, which is comparable with the voltage shift evaluated from the *C*–*V* curve.

The obtained experimental results support in good agreement the predicted course and model simulations of the *C*–*V* curves and ConCap signals of the AuNP-decorated EISCAPs. Thus, in addition to the NP charge [28], the NP coverage significantly affects the performance of the NP-decorated EISCAP biosensors. At the same time, the model enables a predictive comparison to the measurement data, having an additional control for the performed experiments.

## 5. Conclusions

The possibility of gating field-effect EISCAP sensors by the charge of AuNP/molecule hybrids is a very promising strategy for label-free biosensing. In this work, we developed the electrical equivalent circuit and a capacitive model for the EISCAPs decorated with ligand-stabilized charged AuNPs. In this model, AuNPs are considered as tunable nanometer-sized local gates, while the EISCAP is assumed to consist of two regions: an AuNP-covered and an AuNP-free. The *C*–*V* curves and ConCap signal changes of the AuNP-decorated EISCAPs have been simulated as a function of the AuNP coverage.

To determine the impact of the AuNP coverage on the shift of the *C*–*V* curves and ConCap signals experimentally, EISCAPs structures (Al–p-Si–SiO_2_) were decorated with different coverages of positively charged aminooctanethiol-capped AuNPs. As predicted by the theoretical model, the amplitude of the gate-voltage shift increases with increasing the AuNP coverage. A ConCap-signal shift of about −30 mV was registered at a maximum AuNP coverage of *n* = 0.36, which was achieved in this study. The simulations and experimental results indicate, that in addition to the AuNP charge, the surface coverage of AuNPs may significantly influence the EISCAP signal and therefore, the performance of the AuNP-decorated EISCAP sensors.

While the model developed in this study describes the particular case of AuNP-decorated EISCAPs, it can be also be extended for EISCAPs modified with other charged nano-objects, such as ligand-capped magnetic or oxide particles, carbon nanotubes and virus particles. Moreover, the model can help to interpret the changes in the *C*–*V* curves and ConCap signals of EISCAP biosensors caused by biorecognition events between charged target molecules and receptors that are immobilized on the nanoparticles. Finally, the fundamental assumption of the present work, that charged NPs act as nanometer-sized local gates, may be transferred to other types of field-effect sensors such as ISFETs, silicon nanowire transistors or light-addressable potentiometric sensors.

## Data Availability

The data presented in this study are available on request from the corresponding author.

## References

[1] Vu C.-A., Chen W.-Y. (2019). Field-effect transistor biosensors for biomedical applications: Recent advances and future prospects. Sensors.

[2] Syu Y.-C., Hsu W.-E., Lin C.-T. (2018). Field-effect transistor biosensing: Devices and clinical applications. ECS J. Solid State Sci. Technol..

[3] Sakata T. (2019). Biologically coupled gate field-effect transistors meet in vitro diagnostics. ACS Omega.

[4] Panahi A., Sadighbayan D., Forouhi S., Ghafar-Zadeh E. (2021). Recent advances of field-effect transistor technology for infectious diseases. Biosensors.

[5] Poghossian A., Jablonski M., Molinnus D., Wege C., Schöning M.J. (2020). Field-effect sensors for virus detection: From Ebola to SARS-CoV-2 and plant viral enhancers. Front. Plant Sci..

[6] Poghossian A., Schöning M.J. (2020). Capacitive field-effect EIS chemical sensors and biosensors: A status report. Sensors.

[7] Sarcina L., Macchia E., Tricase A., Scandurra C., Imbriano A., Torricelli F., Cioffi N., Torsi L., Bollella P. (2022). Enzyme based field effect transistor: State-of-the-art and future perspectives. ELSA.

[8] Poghossian A., Schöning M.J. (2021). Recent progress in silicon-based biologically sensitive field-effect devices. Curr. Opin. Electrochem..

[9] Cao S., Sun P., Xiao G., Tang Q., Sun X., Zhao H., Zhao S., Lu H., Yue Z. (2022). ISFET-based sensors for (bio)chemical applications: A review. ELSA.

[10] Andrianova M.S., Kuznetsov E.V., Grudtsov V.P., Kuznetsov A.E. (2018). CMOS-compatible biosensor for L-carnitine detection. Biosens. Bioelectron..

[11] Jablonski M., Münstermann F., Nork J., Molinnus D., Muschallik L., Bongaerts J., Wagner T., Keusgen M., Siegert P., Schöning M.J. (2021). Capacitive field-effect biosensor applied for the detection of acetoin in alcoholic beverages and fermentation broths. Phys. Status Solidi A.

[12] Lin C.F., Kao C.H., Lin C.Y., Chen K.L., Lin Y.H. (2020). NH_3_ plasma-treated magnesium doped zinc oxide in biomedical sensors with electrolyte-insulator-semiconductor (EIS) structure for urea and glucose applications. Nanomaterials.

[13] Molinnus D., Beging S., Lowis C., Schöning M.J. (2020). Towards a multi-enzyme capacitive field-effect biosensor by comparative study of drop-coating and nano-spotting technique. Sensors.

[14] Abouzar M.H., Poghossian A., Siqueira J.R., Oliveira O.N., Moritz W., Schöning M.J. (2010). Capacitive electrolyte-insulator-semiconductor structures functionalised with a polyelectrolyte/enzyme multilayer: New strategy for enhanced field-effect biosensing. Phys. Status Solidi A.

[15] Keeble L., Moser N., Rodriguez-Manzano J., Georgiou P. (2020). ISFET-based sensing and electric field actuation of DNA for on-chip detection: A review. IEEE Sens. J..

[16] Abouzar M.H., Poghossian A., Cherstvy A.G., Pedraza A.M., Ingebrandt S., Schöning M.J. (2012). Label-free electrical detection of DNA by means of field-effect nanoplate capacitors: Experiments and modeling. Phys. Status Solidi A.

[17] Bronder T.S., Jessing M.P., Poghossian A., Keusgen M., Schöning M.J. (2018). Detection of PCR-amplified tuberculosis DNA fragments with polyelectrolyte-modified field-effect sensors. Anal. Chem..

[18] Veigas B., Fortunato E., Baptista P.V. (2015). Field effect sensors for nucleic acid detection: Recent advances and future perspectives. Sensors.

[19] Vozgirdaite D., Ben Halima H., Bellagambi F.G., Alcacer A., Palacio F., Jaffrezic-Renault N., Zine N., Bausells J., Elaissari A., Errachid A. (2021). Development of an ImmunoFET for analysis of tumour necrosis factor-α in artificial saliva: Application for heart failure monitoring. Chemosensors.

[20] Kutovyi Y., Li J., Zadorozhnyi I., Hlukhova H., Boichuk N., Yehorov D., Menger M., Vitusevich S. (2020). Highly sensitive and fast detection of C-reactive protein and troponin biomarkers using liquidgated single silicon nanowire biosensors. MRS Adv..

[21] Sinha A., Tai T.-Y., Li K.-H., Gopinathan P., Chung Y.-D., Sarangadharan I., Ma H.-P., Huang P.-C., Shiesh S.-C., Wang Y.-L. (2019). An integrated microfluidic system with field-effect-transistor sensor arrays for detecting multiple cardiovascular biomarkers from clinical samples. Biosens. Bioelectron..

[22] Rani D., Pachauri V., Madaboosi N., Jolly P., Vu X.-T., Estrela P., Chu V., Conde J.P., Ingebrandt S. (2018). Top-down fabricated silicon nanowire arrays for field-effect detection of prostate-specific antigen. ACS Omega.

[23] Si K., Cheng S., Hideshima S., Kuroiwa S., Nakanishi T., Osaka T. (2018). Multianalyte detection of cancer biomarkers in human serum using a label-free field effect transistor biosensor. Sens. Mater..

[24] Bronder T.S., Poghossian A., Jessing M.P., Keusgen M., Schöning M.J. (2019). Surface regeneration and reusability of label-free DNA biosensors based on weak polyelectrolyte-modified capacitive field-effect structures. Biosens. Bioelectron..

[25] Garyfallou G.Z., De Smet L.C., Sudhölter E.J. (2012). The effect of the type of doping on the electrical characteristics of electrolyte–oxide–silicon sensors: pH sensing and polyelectrolyte adsorption. Sens. Actuators B Chem..

[26] Gun J., Rizkov D., Lev O., Abouzar M.H., Poghossian A., Schöning M.J. (2009). Oxygen plasma-treated gold nanoparticle-based field-effect devices as transducer structures for bio-chemical sensing. Microchim. Acta.

[27] Karschuck T., Kaulen C., Poghossian A., Wagner P.H., Schöning M.J. (2021). Gold nanoparticle-modified capacitive field-effect sensors: Studying the surface density of nanoparticles and coupling of charged polyelectrolyte macromolecules. ELSA.

[28] Poghossian A., Bäcker M., Mayer D., Schöning M.J. (2015). Gating capacitive field-effect sensors by the charge of nanoparticle/molecule hybrids. Nanoscale.

[29] Jablonski M., Poghossian A., Severins R., Keusgen M., Wege C., Schöning M.J. (2021). Capacitive field-effect biosensor studying adsorption of *tobacco mosaic virus* particles. Micromachines.

[30] Hideshima S., Hayashi H., Hinou H., Nambuya S., Kuroiwa S., Nakanishi T., Momma T., Nishimura S.-I., Sakoda Y., Osaka T. (2019). Glycan-immobilized dual-channel field effect transistor biosensor for the rapid identification of pandemic influenza viral particles. Sci. Rep..

[31] Lee N., Hyeon T. (2012). Designed synthesis of uniformly sized iron oxide nanoparticles for efficient magnetic resonance imaging contrast agents. Chem. Soc. Rev..

[32] Katz E., Poghossian A., Schöning M.J. (2017). Enzyme-based logic gates and circuits-analytical applications and interfacing with electronics. Anal. Bioanal. Chem..

[33] Poghossian A., Malzahn K., Abouzar M.H., Mehndiratta P., Katz E., Schöning M.J. (2011). Integration of biomolecular logic gates with field-effect transducers. Electrochim. Acta.

[34] Poghossian A., Lüth H., Schultze J., Schöning M. (2001). (Bio-)chemical and physical microsensor arrays using an identical transducer principle. Electrochim. Acta.

[35] Ding S., Cargill A.A., Medintz I.L., Claussen J.C. (2015). Increasing the activity of immobilized enzymes with nanoparticle conjugation. Curr. Opin. Biotechnol..

[36] Chen M., Zeng G., Xu P., Lai C., Tang L. (2017). How do enzymes ‘meet’ nanoparticles and nanomaterials?. Trends. Biochem. Sci..

[37] Ansari S.A., Husain Q. (2012). Potential applications of enzymes immobilized on/in nano materials: A review. Biotechnol. Adv..

[38] Chand R., Han D., Neethirajan S., Kim Y.-S. (2017). Detection of protein kinase using an aptamer on a microchip integrated electrolyte-insulator-semiconductor sensor. Sens. Actuators B Chem..

[39] Xue Q., Bian C., Tong J., Sun J., Zhang H., Xia S. (2012). FET immunosensor for hemoglobin A1c using a gold nanofilm grown by a seed-mediated technique and covered with mixed self-assembled monolayers. Microchim. Acta.

[40] Presnova G., Presnov D., Krupenin V., Grigorenko V., Trifonov A., Andreeva I., Ignatenko O., Egorov A., Rubtsova M. (2017). Biosensor based on a silicon nanowire field-effect transistor functionalized by gold nanoparticles for the highly sensitive determination of prostate specific antigen. Biosens. Bioelectron..

[41] Yang H., Sakata T. (2019). Molecular-charge-contact-based ion-sensitive field-effect transistor sensor in microfluidic system for protein sensing. Sensors.

[42] Siqueira J.R., Maki R.M., Paulovich F.V., Werner C.F., Poghossian A., de Oliveira M.C.F., Zucolotto V., Oliveira O.N., Schöning M.J. (2010). Use of information visualization methods eliminating cross talk in multiple sensing units investigated for a light-addressable potentiometric sensor. Anal. Chem..

[43] Bougrini M., Baraket A., Jamshaid T., Aissari A.E., Bausells J., Zabala M., Bari N.E., Bouchikhi B., Jaffrezic-Renault N., Abdelhamid E. (2016). Development of a novel capacitance electrochemical biosensor based on silicon nitride for ochratoxin A detection. Sens. Actuators B Chem..

[44] Poghossian A., Jablonski M., Koch C., Bronder T.S., Rolka D., Wege C., Schöning M.J. (2018). Field-effect biosensor using virus particles as scaffolds for enzyme immobilization. Biosens. Bioelectron..

[45] Yang C.-F., Hwu J.-G. (2016). Role of fringing field on the electrical characteristics of metal-oxide-semiconductor capacitors with co-planar and edge-removed oxides. AIP Adv..

[46] Poghossian A., Welden R., Buniatyan V.V., Schöning M.J. (2021). An array of on-chip integrated, individually addressable capacitive field-effect sensors with control gate: Design and modelling. Sensors.

[47] Fabry P., Laurent-Yvonnou L. (1990). The C-V method for characterizing ISFET or EOS devices with ion-sensitive membranes. J. Electroanal. Chem. Interfacial Electrochem..

[48] Sze S.M., Ng K.K. (2006). Physics of Semiconductor Devices.

[49] Polte J. (2015). Fundamental growth principles of colloidal metal nanoparticles—A new perspective. CrystEngComm.

[50] Wuithschick M., Birnbaum A., Witte S., Sztucki M., Vainio U., Pinna N., Rademann K., Emmerling F., Kraehnert R., Polte J. (2015). Turkevich in new robes: Key questions answered for the most common gold nanoparticle synthesis. ACS Nano.

[51] Kaulen C., Homberger M., Bourone S., Babajani N., Karthäuser S., Besmehn A., Simon U. (2014). Differential adsorption of gold nanoparticles to gold/palladium and platinum surfaces. Langmuir.

[52] Calculating Size Distribution of Powder Particles Using ImageJ. https://www.fzu.cz/~dominecf/index.html.

[53] Ben Haddada M., Huebner M., Casale S., Knopp D., Niessner R., Salmain M., Boujday S. (2016). Gold nanoparticles assembly on silicon and gold surfaces: Mechanism, stability, and efficiency in diclofenac biosensing. J. Phys. Chem. C.

[54] Aureau D., Varin Y., Roodenko K., Seitz O., Pluchery O., Chabal Y.J. (2010). Controlled deposition of gold nanoparticles on well-defined organic monolayer grafted on silicon surfaces. J. Phys. Chem. C.

[55] Lowe B.M., Sun K., Zeimpekis I., Skylaris C.-K., Green N.G. (2017). Field-effect sensors—From pH sensing to biosensing: Sensitivity enhancement using streptavidin-biotin as a model system. Analyst.

[56] De Moraes A., Kubota L. (2016). Recent trends in field-effect transistors-based immunosensors. Chemosensors.

[57] Bhattacharyya I.M., Ron I., Chauhan A., Pikhay E., Greental D., Mizrahi N., Roizin Y., Shalev G. (2022). A new approach towards the Debye length challenge for specific and label-free biological sensing based on field-effect transistors. Nanoscale.

[58] Cloarec J.-P., Chevalier C., Genest J., Beauvais J., Chamas H., Chevolot Y., Baron T., Souifi A. (2016). pH driven addressing of silicon nanowires onto Si_3_N_4_/SiO_2_ micro-patterned surfaces. Nanotechnology.

[59] Goyne K.W., Zimmerman A.R., Newalkar B.L., Komarneni S., Brantley S.L., Chorover J. (2002). Surface charge of variable porosity Al_2_O_3_(s) and SiO_2_(s) adsorbents. J. Porous Mater..

[60] Bousse L., Mostarshed S., van der Shoot B., De Rooij N.F., Gimmel P., Göpel W. (1991). Zeta potential measurements of Ta_2_O_5_ and SiO_2_ thin films. J. Colloid Interface Sci..

